# Elevation of glycoprotein nonmetastatic melanoma protein B in type 1 Gaucher disease patients and mouse models

**DOI:** 10.1002/2211-5463.12078

**Published:** 2016-07-30

**Authors:** Gertjan Kramer, Wouter Wegdam, Wilma Donker‐Koopman, Roelof Ottenhoff, Paulo Gaspar, Marri Verhoek, Jessica Nelson, Tanit Gabriel, Wouter Kallemeijn, Rolf G. Boot, Jon D. Laman, Johannes P.C. Vissers, Timothy Cox, Elena Pavlova, Mary Teresa Moran, Johannes M. Aerts, Marco van Eijk

**Affiliations:** ^1^ Department of Medical BiochemistryAcademic Medical CenterAmsterdamThe Netherlands; ^2^ European Molecular Biology LaboratoryGermany; ^3^ Department of GynecologyAcademic Medical CenterAmsterdamThe Netherlands; ^4^ Organelle Biogenesis & Function GroupInstituto de Investigação e Inovação em Saúde (I3S)PortoPortugal; ^5^ Institute of Molecular and Cell Biology (IBMC)Universidade do PortoPortugal; ^6^ Instituto de Ciências Biomédicas Abel Salazar (ICBAS)Universidade do PortoPortugal; ^7^ Department of Medical BiochemistryLeiden Institute of ChemistryLeiden UniversityThe Netherlands; ^8^ Department of NeuroscienceUniversity Medical Center GroningenThe Netherlands; ^9^ Waters CorporationWilmslowUK; ^10^ Department of Internal MedicineAddenbrooke's HospitalCambridgeUK

**Keywords:** chitotriosidase, DC‐HIL, glucosylceramide, lysosome, osteoactivin, storage disease

## Abstract

Gaucher disease is caused by inherited deficiency of lysosomal glucocerebrosidase. Proteome analysis of laser‐dissected splenic Gaucher cells revealed increased amounts of glycoprotein nonmetastatic melanoma protein B (gpNMB). Plasma gpNMB was also elevated, correlating with chitotriosidase and CCL18, which are established markers for human Gaucher cells. In Gaucher mice, gpNMB is also produced by Gaucher cells. Correction of glucocerebrosidase deficiency in mice by gene transfer or pharmacological substrate reduction reverses gpNMB abnormalities. In conclusion, gpNMB acts as a marker for glucosylceramide‐laden macrophages in man and mouse and gpNMB should be considered as candidate biomarker for Gaucher disease in treatment monitoring.

**Abbreviations**
DC‐HIL: DC‐associated heparan sulfate proteoglycan‐dependent integrin ligand
ERT: enzyme replacement therapy
GBA: glucocerebrosidase
GD: Gaucher Disease
gpNMB: glycoprotein nonmetastatic melanoma protein B
NPC: Niemann–Pick type C
PGK: phosphoglycerate kinase
SRT: substrate reduction

The common lysosomal storage disorder Gaucher disease (GD) is an autosomal recessively inherited deficiency of lysosomal glucocerebrosidase (GBA; EC 3.2.1.45). GD is clinically heterogeneous, ranging from the most common non‐neuronopathic variant (type 1) to more severe manifestations involving the central nervous system and skin 1. Common to all GD variants is accumulation of glucosylceramide in lysosomes of tissue macrophages causing their transformation to characteristic lipid‐laden Gaucher cells 1. Progressive accumulation of Gaucher cells in several organs like spleen, liver, bone marrow, and lung is thought to lead to the spectrum of visceral symptoms such as hepatosplenomegaly, pancytopenia, and bone disease 1. Monitoring of factors released by Gaucher cells in blood allows detection of actual progression of storage cell formation in patients, a highly variable process that is not predicted well by the GBA genotype as most clearly illustrated by discordant phenotypes in monozygotic GD twins 2, 3, 4. In addition, plasma markers of Gaucher cells also offer guidance for decisions on initiation of therapy and individualized dosing regimens 5. We previously discovered that Gaucher cells massively secrete chitotriosidase, an around 1000‐fold elevated enzyme in plasma of symptomatic type 1 GD patients 6. As sensitive biomarker of Gaucher cells, it is currently used for diagnosis, early disease onset, and therapeutic efficacy monitoring 2, 6, 7. Chitotriosidase does not reflect one particular clinical symptom, but rather the total body burden of Gaucher cells 7. Its use as a marker is hampered by the fact that about 5% to 6% of individuals, including GD patients, are deficient in chitotriosidase activity 8. These individuals cannot be monitored via chitotriosidase activity. Other common polymorphisms in chitotriosidase affecting substrate affinity, limit interpretation of data 9. The chemokine CCL18, also named PARC, is overexpressed by Gaucher cells as well. In plasma and urine of symptomatic GD patients, several 10‐fold elevations exist offering a good alternative to monitor storage cells 10, 11, 12. Recently, mouse models representing type 1 GD pathology have been generated using inducible deficiency in GBA in the white blood cell lineage 13, 14. The characteristic lipid‐laden macrophages develop in spleen, liver and bone marrow, recapitulating the human disease. The promoter of the mouse chitotriosidase gene is fundamentally different not driving phagocyte‐specific expression 15, while the CCL18 gene is entirely absent in the mouse genome 16.

To identify additional surrogate markers of Gaucher cells in man and mouse, we investigated the proteome of laser‐dissected storage cells from spleen of a type 1 GD patient. gpNMB was prominently detected, is expressed in macrophages, can be released as a soluble fragment upon lysosomal stress, has earlier been connected to the disease, and very recently increased levels were reported in cerebrospinal fluid of the neuropathic form of GD 7, 17, 18. Hence, we investigated gpNMB in detail. First, soluble gpNMB is markedly increased in plasma of symptomatic type 1 GD patients. Second, gpNMB correlates with chitotriosidase and CCL18 in plasma, prior and during therapy. Finally, GBA‐deficient mice developing lipid‐laden macrophages also show increased gpNMB in tissues and circulation. In conclusion, gpNMB has potential value as a surrogate marker for GD in man and mouse models.

## Materials and methods

### Patients

All 59 type 1 Gaucher patients studied (30 males and 29 females) were known by referral to the Academic Medical Center. The type I Gaucher disease patients studied were 15–64 years old at the initiation of therapy (*t* = 0). gpNMB levels were analyzed prior to and 1 year after initiation of enzyme replacement therapy. Diagnosis was confirmed by demonstration of markedly reduced glucocerebrosidase activity and mutant GBA genotype. The controls consisted of 10 male and 10 female healthy volunteers. Plasma samples were collected prior to the start of enzyme replacement therapy. Approval was obtained from the Ethics Committee of the Academic Medical Center, Amsterdam. Informed consent was provided according to the Declaration of Helsinki.

### Mouse materials

The generation of the GD1 mouse model (Gbatm1Karl/tm1KarlTg(Mx1‐cre)1Cgn/0) with inducible knock down of GBA in the white blood cell lineage has been described previously 13. Materials, kindly provided by Karlsson (Lund), were analyzed previously for other biochemical parameters 19. *Scarb2* ^−/−^ mice deficient in LIMP2 were kindly provided by Saftig (Kiel) 20. The mice were housed at the Academic Medical Center Institute Animal Core Facility in a temperature‐ and humidity‐controlled room with a 12‐h light/dark cycle and given free access to food and water ad libitum. All animal protocols were approved by the Institutional Animal Welfare Committee of the Academic Medical Centre Amsterdam in the Netherlands. Mice were sacrificed according to protocol, being first anesthetized with a dose of Hypnorm (0.315 mg·mL^−1^ phenyl citrate and 10 mg·mL^−1^ fluanisone) and Dormicum (5 mg·mL^−1^ midazolam) according to their weight. The administered dose was 80 μL per 10 g body weight. Anesthetized animals were sacrificed by cervical dislocation. Organs were collected by surgery and rinsed with PBS. All samples and tissues collected were stored at −80 °C prior to further analysis.

### Macrophages

Monocytes were cultured for up to 10 days in RPMI 1640 (BioWhittaker, Viviers, Belgium) supplemented with 10% human serum (BioWhittaker). Phenotypical maturation was confirmed by light microscopy. RIPA (150 mmol·L^−1^ NaCl, 50 mmol·L^−1^ Tris‐HCl pH 7.4, 2 mmol·L^−1^ EDTA, 0.5% deoxycholaat, 1 mmol·L^−1^ Na_3_VO_4_, 20 mmol·L^−1^ NaF, and 0.5% Triton X‐100) supplemented with protease inhibitor cocktail (Roche, Almere, the Netherlands) and PMSF lysates were prepared directly after isolation and after 1, 3, 5, 7, and 10 days of culture.

### Enzyme activity assays

The enzymatic activity assay for chitotriosidase with 4 MU‐deoxychitobiose as substrate was performed at pH 5.2, as described previously 21.

### Glucosylsphingosine measurement

The glycosphingoid base glucosylsphingosine was measured as described earlier 19.

### ELISA and western blot

Plasma gpNMB levels were measured using commercially available ELISAs as described by the manufacturer (R&D systems, Abingdon, UK). Western blotting of gpNMB was performed using commercial mouse anti‐gpNMB antibody (MAB25501; R&D systems). Plasma CCL18 concentrations were determined by ELISA exactly as described earlier 10.

### RNA isolation, northern blot, and qPCR

Total spleen RNA of a type 1 patient was isolated using the RNAzol B (Biosolve, Barneveld, The Netherlands) RNA isolation kit according to the manufacturer's instructions. For Northern blot analysis, 15‐μg samples of total RNA were run in 10 mm HEPES (*N*‐2‐hydroxyethylpiperazine‐*N*’‐2‐ethanesulfonic acid), 6% formaldehyde‐agarose gels, transferred to Hybond N nylon membranes (Amersham, Buckinghamshire, UK) by the capillary method, and immobilized by UV cross‐linking. The following probes were used: full‐length gpNMB cDNA and glyceraldehyde‐3‐phosphate dehydrogenase (GAPDH) as an RNA control. The probes were radiolabeled with ^32^P using the random priming method. qPCR was performed using human gpNMB forward primers 5′‐gggtctgggacgtactgtgt‐3′and reverse primers 5′‐ctcaggcctttgcttctgac‐3′. Expression levels were normalized to those of acidic ribosomal phosphoprotein 36B4, also referred to as P0. Forward 5′‐tcgacaatggcagcatctac‐3′ and reverse 5′‐atccgtctccacagacaagg‐3′.

### Immunohistochemistry

Immunohistochemistry was performed on frozen sections of a type 1 GD spleen to detect expression patterns of gpNMB using an earlier described methodology 22. In brief, frozen sections of 6 μm were cut and thaw‐mounted on glass slides. Slides were kept overnight at RT in humidified atmosphere. After air‐drying the slides for 1 h, slides were fixed in fresh acetone containing 0.02% (vol/vol) H_2_O_2_. Slides were then air‐dried for 10 min, washed with phosphate‐buffered saline (PBS), and incubated with optimally diluted mouse antihuman gpNMB monoclonal antibody (MAB25501; R&D systems) overnight at 4 °C in a humidified atmosphere. Incubations with secondary rabbit anti‐mouse‐Ig‐biotin (Dako, Glostrup, Denmark) and tertiary HRP‐labeled avidin‐biotin‐complex (ABC/HRP; Dako) were performed for 1 h at RT. Between incubation steps, slides were washed twice with PBS. HRP activity was revealed by incubation for 10 min at RT with 3‐amino‐9‐ethyl‐carbazole (AEC; Sigma, Zwijndrecht, the Netherlands), leading to a bright red precipitate. After washing, sections were counterstained with hematoxylin and embedded with glycerol‐gelatin. Primary antibody reagent omission control staining was performed.

### Laser microdissection of Gaucher cells

From frozen spleen of a type 1 patient stored at −80 °C, 10‐μm cryostat sections were prepared. One section was stained with hematoxylin and examined microscopically in order to detect tissue areas of interest for microdissection. Corresponding consecutive tissue sections were mounted on a microscope slide coated with a membrane [polyethylene naphthalate (PEN) Zeiss/Palm, Bernried, Germany] and stored at −80 °C. Comparison of stained and unstained tissue sections revealed that hematoxylin staining had no influence on the quantitative protein measurements and identification using LC‐MS^e^. Tissue areas were cut using a Veritas™ Microdissection System (Arcturus Molecular Devices, CA, USA), as described earlier 23. Slides were stained for 1 min with hematoxylin. The dissected tissue samples contained at least 90% macrophages based on microscopic examination, a few hundred cells from each sample.

### Proteome analysis

Cells were denatured in 20 μL 0.1% RapiGest detergent solution (Waters Corp., Milford, MA, USA) and heated at 80 °C for 15 minutes. After centrifugation at 1750 ***g*** for 10 min, the supernatants were collected. Several tests were performed to determine the optimal lysis conditions. In addition, a small piece of frozen Gaucher or control spleen was homogenized in 30 μL water (HPLC‐grade, Biosolve, The Netherlands) and debris was removed by centrifugation. The protein concentration of the different homogenates was determined by bicinchoninic acid‐assay according to the manufacturer's protocol (Thermo‐scientific, Landsmeer, the Netherlands). Subsequently, Rapigest SF was added to the whole‐spleen homogenates to a final concentration of 0.1% and ammonium bicarbonate to all homogenates to a final concentration of 50 mM. Protein disulfide bridges were reduced and alkylated by subsequent incubation with 10 mM dithiothreitol and 25 mM iodoaceteamide. Tryptic digestion was initiated by the addition of trypsin (gold mass spectrometry grade, Promega, Leiden, the Netherlands) in a ratio by weight of 1:100 (protease: protein) and overnight incubation at 37 °C. Following digestion, breakdown of the acid‐labile Rapigest SF was achieved by addition of trifluoroacetic acid according to the manufacturer's protocol. The protein composition was analyzed by LC‐MS^E^, a mass spectrometric procedure allowing identification and quantification of proteins without the need of labeling 24. For this purpose, samples were centrifuged for 10 min at 14000 ***g*** and ~0.5 μg was loaded onto a NanoAcquity system (Waters Corporation) equipped with a Bridged Ethyl Hybrid C18 1.7 μm, 15‐cm × 150‐μm analytical reversed phase column (Waters Corporation), and operated at a column flow rate of 1 μL·min^−1^ and separated by gradient conditions as described before 24. Analysis of tryptic peptides was performed using a Synapt MS mass spectrometer (Waters Corporation, Manchester, UK) with the operating and experimental conditions as previously described 24 **.** Accurate mass precursor and fragment ion LC‐MS data were collected in data‐independent LC‐MS^E^ mode of acquisition 25, 26. Continuum LC‐MS data were processed and searched using Protein Lynx Global SERVER version 2.3 (Waters Corporation) with search parameters as described before 24 **.** Briefly, tryptic cleavage rules were applied, allowing for 1 missed cleavage, in addition, carbomidomethylation was set as fixed modification of cysteine residues while methionine oxidation was set as variable modification. Protein identifications were obtained searching the human SwissProt entries of a UniProt database (release 13.2) that was modified to include N‐terminal processing of proteins using the protein maturation device software 27 while estimations of false positive rates of protein identifications were obtained by simultaneous searches in a reversed version of the same database generated by ProteinLynx GlobalSERVER. Absolute amounts (ng) of proteins were estimated using the combined intensity of the 3 best ionizing peptides and the universal response factor of the mass spectrometer supplied by the manufacturer 28. Data were exported as a csv‐file for further analysis. Robust criteria were applied for quantification: patient and control samples were run in triplicate and only proteins that were identified in more than one run were reported. False‐positive identification rates per protein were calculated taking into account the criteria mentioned above and were ~0.38%. In order to normalize for differences in protein concentration between samples loaded, each run was normalized on total protein amount based on the bicinchoninic acid‐assay performed on each homogenate. The resulting value reported is ng of protein per μg of total protein (ng·μg^−1^). Proteins that were only identified in either Gaucher or control samples were required to be detected in all three replicate runs, and to be at least 10 times above the signal intensity of the least abundant protein detected in order to be reported.

### Statistical analysis

Correlations were tested by the rank correlation test (Spearman coefficient, rho). *P* values < 0.05 were considered statistically significant.

## Results

### Proteomics of Gaucher cells in GD spleen

**Figure 1:**
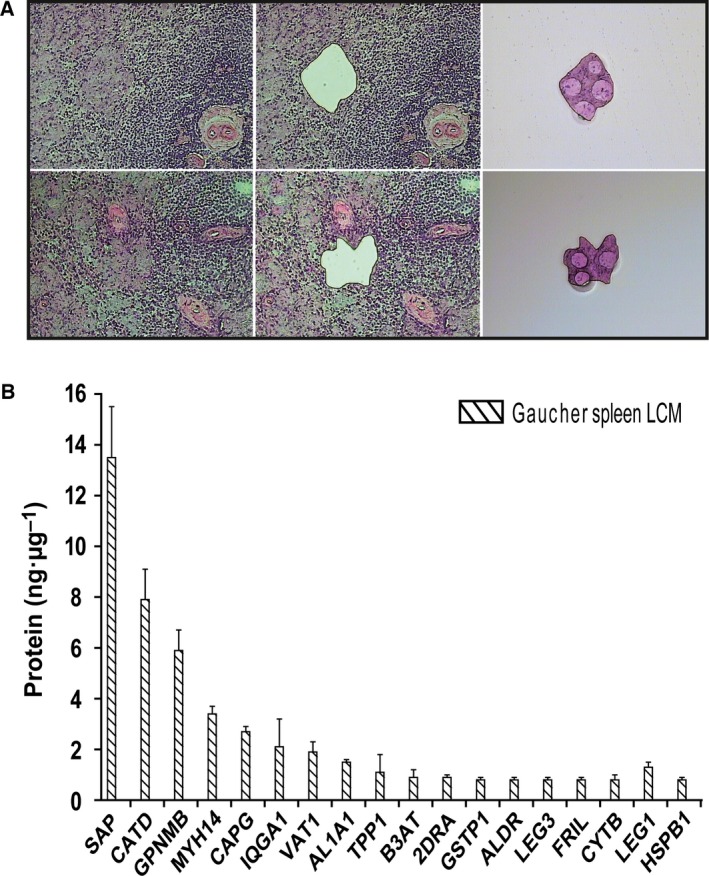
Proteomics of laser‐dissected Gaucher cells. (A) Laser‐dissection of Gaucher cells from type 1 GD spleen. (B) LC‐MS^E^ of isolated Gaucher cells.

To identify novel factors released by Gaucher cells in plasma, we first investigated the protein composition of these cells by mass spectrometry. Lipid‐laden storage cells were isolated from a section of spleen from a type 1 GD patient by means of laser‐dissection (Fig. 1A). The protein composition of the homogenate of pooled sections (*n* = 6), representing approximately 2000 storage cells, was analyzed by LC‐MS^E^ and compared to a similar‐sized fraction of normal spleen. Figure 1B shows that some proteins were highly abundant in storage cell preparations. Most striking in this respect were prosaposin, cathepsin D, and gpNMB. Similar results were obtained following LC‐MS^E^ analysis of total homogenates of GD and control spleens (Fig. S1).

### Elevated gpNMB in GD spleen

**Figure 2:**
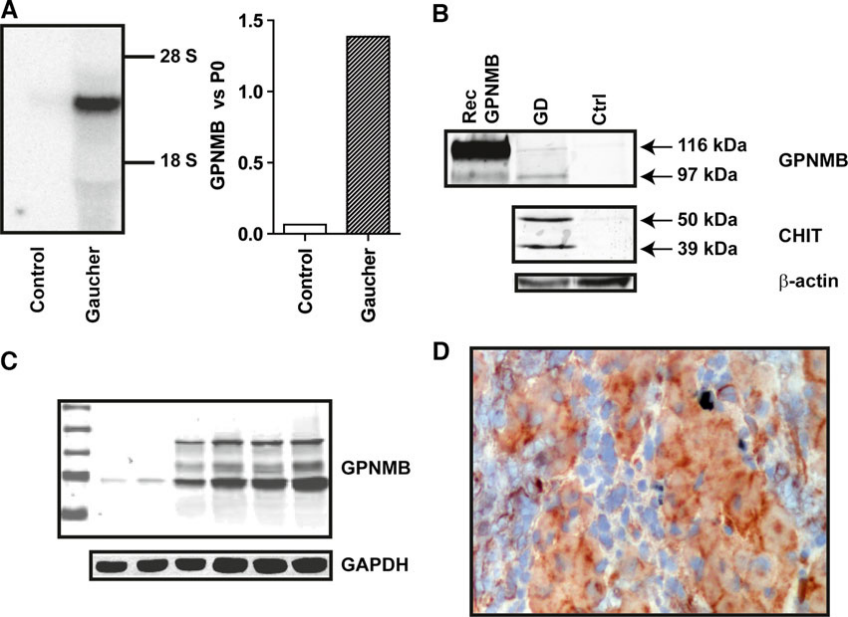
Increased gpNMB in GD spleen. (A) Left panel: northern blot for gpNMB RNA in type 1 GD and control spleen. Right panel: qPCR of gpNMB mRNA in type 1 GD and normal spleen. (B) Western blot detection of gpNMB and chitotriosidase in type 1 GD and control spleen. (C) Western blot detection of gpNMB in human monocyte‐derived macrophages (from left to right marker, monocytes and 1, 3, 5, 7, or, 10 days matured macrophages). (D) Immunohistochemical detection of gpNMB in Gaucher cells in type 1 GD spleen using a mouse monoclonal antibody.

Previous subtractive hybridization studies on GD spleen 11 and microarray analysis (J.M. Aerts, unpublished findings) already pointed to increased expression of gpNMB in type 1 GD spleens. In addition, it has been documented that gpNMB can be shed as a soluble factor 17. The highly increased presence of gpNMB RNA in type 1 GD spleen was demonstrated by Northern blot and qPCR (Fig. 2A). Next, we investigated gpNMB at the protein level. Western blot analysis using an anti‐human gpNMB mouse monoclonal antibody confirmed the proteomics findings (Fig. 2B). In a homogenate of GD spleen, but not that of control tissue, gpNMB was detected. A similar observation was made for chitotriosidase (Fig. 2B). Consistent with high expression of gpNMB in Gaucher cells, we observed expression in human monocyte‐derived macrophages (Fig. 2C). Immunohistochemistry on sections from spleens of a type 1 GD patient unequivocally revealed the specific expression of gpNMB protein in Gaucher cells (Fig. 2D). Expression of gpNMB was virtually absent in other cells in this spleen section.

### Elevated gpNMB in GD plasma

**Figure 3:**
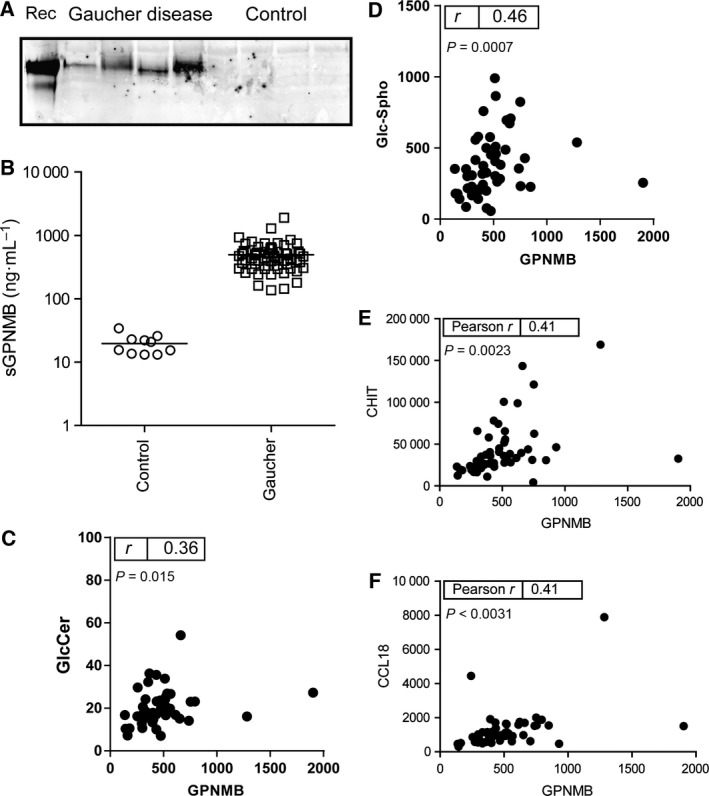
Plasma gpNMB in type 1 GD patients. (A) Western blot detection of gpNMB in 0.5 μL type 1 GD and control plasma. (B) ELISA quantification of gpNMB in type 1 GD (squares) and control plasma (circles). (C) Correlation of plasma gpNMB and glucosylceramide (GlcCer). (D) Correlation of plasma gpNMB and glucosylsphingosine (Glc‐Spho). (E) Correlation of plasma gpNMB and chitotriosidase. (F) Correlation of plasma gpNMB and CCL18.

Since gpNMB can be proteolytically shed from cells, the occurrence of soluble fragments in the circulation was examined. Western blot analysis of plasma (0.5 μL) of type 1 GD patients using anti‐human gpNMB antiserum revealed the presence of gpNMB, but not in the case of plasma of normal subjects (Fig. 3A). To quantify the levels of gpNMB, a sandwich ELISA was used. Figure 3B shows that plasma gpNMB levels were clearly increased (on average 25‐fold) in all tested 59 symptomatic type 1 GD patients (plasma taken prior to initiation of therapy). The mean plasma gpNMB level in controls is 20 ng·mL^−1^ (range, 11–34 ng·mL^−1^), whereas that in patients’ samples was 495 ng·mL^−1^ (range, 137–1283 ng·mL^−1^). No overlap was seen between values in the examined patients and controls. Interestingly, elevated circulatory levels of gpNMB were also observed in three of four type 2/type 3 patients sera analyzed.

The relationship between gpNMB and glucosylceramide and gpNMB and glucosylsphingosine was first determined. Plasma gpNMB correlated with glucosylceramide (ρ = 0.036; *P* = 0.015) and better with glucosylsphingosine (ρ = 0.46; *P* = 0.0007) (Fig. 3C,D). The relationship between gpNMB and two other established Gaucher cell markers, chitotriosidase and CCL18, in plasma of type 1 GD patients was further examined. Plasma gpNMB levels in untreated GD patients correlated well with those of chitotriosidase (ρ = 0.41; *P* < 0.05) and CCL18 (ρ = 0.40; *P* < 0.05) (Fig. 3E,F). A similar correlation was found with total β‐hexosaminidase activity in plasma specimen (ρ = 0.40; *P* < 0.05) (Fig. S2). The correlation of chitotriosidase and CCL18 in the same samples was better (ρ = 0.69; *P* < 0.05) (data not shown). No correlation between plasma gpNMB levels and disease severity was observed.

### Corrections in plasma gpNMB in GD patients receiving therapy

**Figure 4:**
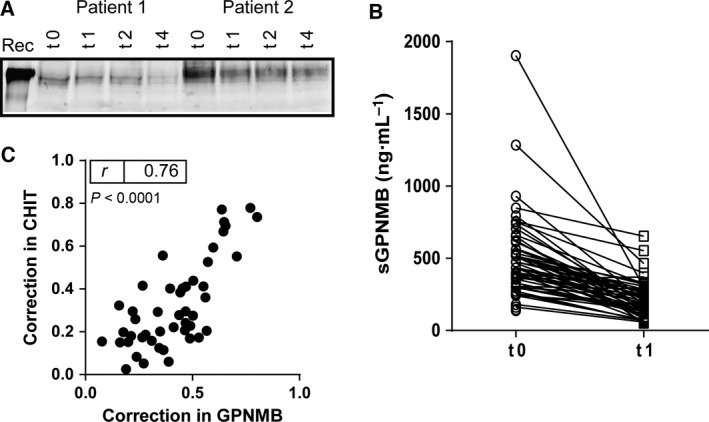
Correction of elevated plasma gpNMB by enzyme replacement therapy. (A) ERT‐induced reduction of plasma gpNMB (western blot). (B) ERT‐induced reductions of plasma gpNMB (ELISA). (C) Correlation of relative change in excess plasma gpNMB and chitotriosidase.

Enzyme replacement therapy (ERT) of type 1 GD patients results in prominent reductions of Gaucher cells in tissues, reflected by corrections in plasma chitotriosidase levels 5. We investigated the effect of ERT on plasma gpNMB of type 1 GD patients (Fig. 4). Western blot analysis of plasma samples collected from patients during therapy already revealed a prominent reduction in gpNMB protein (Fig. 4A). Quantification of plasma gpNMB by ELISA in samples collected at start of ERT and after 1 year treatment (range 10–14 months) showed major correction in plasma gpNMB with treatment in nearly every patient (Fig. 4B and Table S1). We next compared the corrections in plasma gpNMB with those in chitotriosidase (value at start of ERT being 100%). A very strong correlation was observed in relative corrections of gpNMB and chitotriosidase (Fig. 4C). The two most striking responders for gpNMB (84% and 92% reduction) also showed concomitantly a striking reduction in chitotriosidase (both cases 85%), indicating an impressive reduction in Gaucher cells.

### gpNMB in GD mice

**Figure 5:**
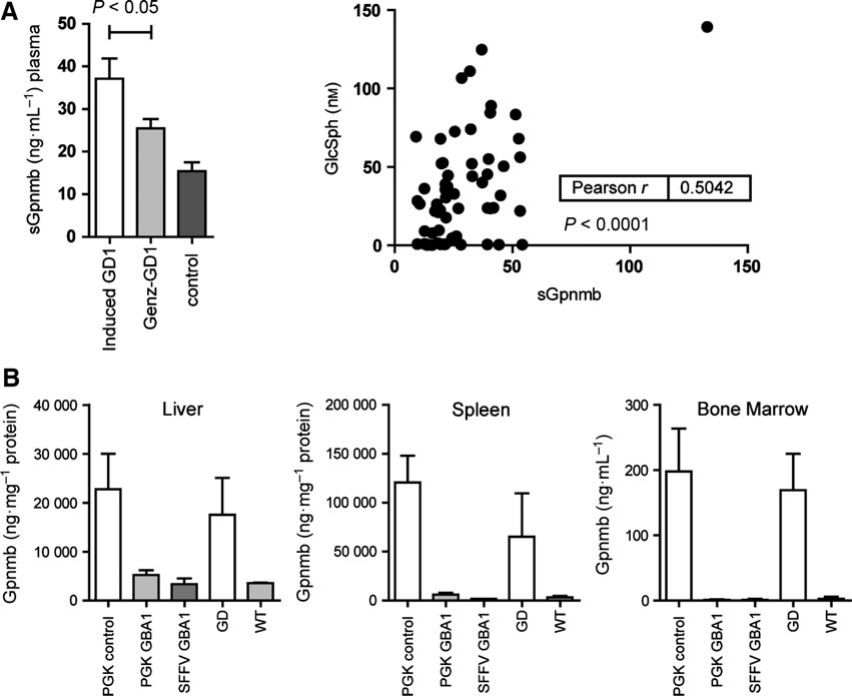
Elevated gpNMB in GD mice. (A) Increased plasma gpNMB in GD mice and effect of substrate reduction therapy using Eliglustat (GENZ) in GD mice (left panel). Data are depicted as mean ± SEM. Correlation of plasma glucosylsphingosine and gpNMB in GD and Eliglustat treated mice (right panel). (B) Effect of GBA gene therapy on gpNMB in mice on liver, spleen, and bone marrow. PGK control indicates treatment with self‐inactivating lentiviral vectors without the GBA gene under the control of human phosphoglycerate kinase (PGK); PCK GBA1: same vector with GBA gene; SFFV GBA1: vector with constitutive SFFV promotor and GBA gene; GD untreated animals; WT: matched normal animals (see ref. 19). Data are depicted as mean ± SEM. *n* = 3 per group.

Next, we examined gpNMB in type 1 GD mice with inducible knock down of glucocerebrosidase in the hematopoietic lineage by polyinosinic‐polycytidylic acid treatment (*Gba* ^*tm1Karl/tm1Karl*^) (Fig. 5). These animals develop characteristic Gaucher cells for instance found in spleen and liver when expression of glucocerebrosidase is down‐regulated 19. The level of gpNMB is markedly increased in plasma of the GD mice (Fig. 5A). The effect of therapeutic interventions on plasma gpNMB was subsequently investigated. We first studied the impact of substrate reduction (SRT) as accomplished by administration of eliglustat, a potent inhibitor of the synthesis of glucosylceramide by GCS and recently registered by the FDA for the treatment of type 1 GD 29, 30. In SRT‐treated type 1 GD mice (*Gba* ^*tm1Karl/tm1Karl*^) plasma gpNMB was reduced (Fig. 5A). The reduction in plasma gpNMB correlated with that of glucosylsphingosine, a measure for the presence of lipid‐laden macrophages 31, 32, 33 (Fig. 5A). Next, we examined the effect of treatment of diseased *Gba* ^*tm1Karl/tm1Karl*^ mice through gene therapy with self‐inactivating lentiviral vectors with the GBA gene under the control of human phosphoglycerate kinase (PGK) as reported previously 19. Mice which were functionally corrected in GBA, showed a reduction of gpNMB in liver, spleen, and bone marrow (Fig. 5B).

Mice with a genetic loss of *SCARB2* lacking the membrane protein LIMP2 that transports GBA to lysosomes show marked GBA deficiency in most tissues and cell types, except white blood cells. The *Scarb2* ^−/−^ mice, like LIMP2 deficient patients, suffer from action myoclonus renal failure syndrome, but do not develop lipid‐laden macrophages as in GD 34, 35. We could not detect abnormalities in gpNMB in LIMP2‐deficient mice (see Fig. S3). This finding illustrates the need of lysosomal stress, hence lysosomal lipid accumulation causing lysosomal perturbations, in macrophages for gpNMB overexpression, and its release in the circulation. In conclusion, our findings indicate that gpNMB may serve as a sensitive marker for formation of lipid‐laden cells in GD mouse models.

## Discussion

This study extends our earlier observation of abnormalities in gpNMB in GD patients 7, 11 toward identification of a candidate biomarker for therapy monitoring. We here describe that in a cohort of symptomatic type 1 GD patients, plasma gpNMB is commonly and markedly increased. We show that gpNMB is produced by lipid‐laden macrophages, Gaucher cells, in tissues of GD patients. gpNMB levels correlate strongly with those of validated Gaucher cell markers such as chitotriosidase and glucosylsphingosine. Changes in Gaucher cell markers in GD patients receiving therapy are accompanied by comparable corrections in gpNMB. Finally, immunohistochemistry of spleen and data for laser‐dissected Gaucher cells confirm overexpression of gpNMB by Gaucher cells. Consistent with our finding, Futerman and colleagues reported very recently elevated levels of gpNMB in cerebrospinal fluid of neuronopathic GD patients as well as neuronopathic GD mice 18. They proposed that gpNMB may serve as a marker to quantify neuropathology. Of note, activated microglia, the CNS functional counterpart of macrophages, recently have been reported to express gpNMB 36. gpNMB plasma levels are also strongly elevated in mouse models of Niemann–Pick type C (NPC) disease and to a lesser degree in NPC patients. NPC‐macrophages generated using the drug U18666A also induced Gpnmb and glycosphingolipid synthesis inhibition prevented this 37. gpNMB is expressed by phagocytic cell types like macrophages, dendritic cells, and osteoclasts as well as melanocytes 17. It has also been documented earlier that breast cancer cells release soluble fragments through active shedding, presumably by the action of the membrane‐bound protease ADAM10 38. We recently reported that gpNMB expression in macrophages is under the control of the transcription factor MITF and is markedly increased upon lysosomal stress 39. Increased GPNMB mRNA was noted previously for type 1 GD spleen 11 and for liver of mice with induced GBA deficiency in hematopoietic and mesenchymal cells 14. All in all, the here documented marked increase in circulating gpNMB in type 1 GD patients now fully documented here is entirely consistent with previous more scattered findings.

Given the marked abnormality in gpNMB in type 1 GD patients, recent insights in the function of this protein warrant discussion. In the previous years, the protein gpNMB, also named osteoactivin or DC‐HIL (DC‐associated heparan sulfate proteoglycan‐dependent integrin ligand), received considerable attention in different research fields. A number of investigators have focused on its role in bone metabolism. The expression of gpNMB in osteoclasts has been documented for some time, but in addition, very recent studies point to a crucial role of osteoactivin in bone formation as indicated by the negative consequences of deficiency of the protein for bone generation 40, 41, 42. It is of interest to note that impaired bone formation is presently considered an important contributor to the complex skeletal abnormalities observed in type 1 GD patients 14, 43. Our findings might therefore stimulate further investigations on the potential role of this protein in skeletal GD disease.

The protein gpNMB has also drawn attention of researchers studying the interaction of dendritic cells (DC) and T cells. gpNMB is expressed by dendritic cells critically regulated by the transcription factor MITF 44. gpNMB on antigen presenting cells inhibits T‐cell activation by binding to syndecan‐4 (SD‐4) on T cells 45, 46. It has been hypothesized that the DC‐HIL/SD‐4 pathway regulates autoimmune responses. Investigations on experimental autoimmune encephalomyelitis (EAE) as a model for multiple sclerosis indeed have rendered evidence that the DC‐HIL/SD‐4 pathway regulates autoimmune responses by mediating the T‐cell suppressor function of CD11b(+)Gr‐1(+) myeloid‐derived suppressor cells (MDSC) 47. Of note, a genome‐wide association meta‐analysis of 12 386 Parkinson disease (PD) cases and 21 026 controls led to the identification of PD risk loci, including gpNMB (7p15) 48, 49. Also from functional studies, in recent years, it has become apparent that also mutations in GBA, the primary defect in GD, constitute a risk for developing PD 50.

Beyond its tentative (patho)physiological functions, gpNMB is of interest as a potential marker for lipid‐laden macrophages in type 1 GD. Immunohistochemical analysis of type 1 GD spleen sections demonstrates that Gaucher cells are a prominent source of gpNMB. The close correlation of plasma gpNMB with two established markers of Gaucher cells, chitotriosidase and CCL18, suggests that it reflects the body burden of Gaucher cells. Additional markers of Gaucher cells are still of value: the validated biomarker chitotriosidase cannot be used in every individual due to gene mutations 7. The plasma concentration of chemokine CCL18 is close to normal in mildly affected type 1 GD patients. In our view, plasma gpNMB can serve as additional GD marker for further confirmation of diagnosis, and monitoring of disease progression or correction following therapeutic intervention. We already noted that fractional corrections in plasma gpNMB and chitotriosidase are similar in type 1 GD patients during therapy. Of note, plasma gpNMB, like chitotriosidase and CCL18, does not reflect one particular clinical manifestation of type 1 GD. We like to stress that measurement of the plasma gpNMB for primary diagnosis of GD should not be advocated. Increased expression has been also noted in other disease conditions, as for example, illustrated by the recent report on atherosclerosis 51. Only in cases where GD has been demonstrated by GBA deficiency and gene defects, monitoring of plasma gpNMB level is useful.

Our investigation further revealed that also in mice, gpNMB is excessively produced and released by glucosylceramide‐laden macrophages with deficient GBA activity. Vice versa, gpNMB abnormalities are reduced by correction of the GBA deficiency or prevention of lysosomal glucosylceramide accumulation. Since chitotriosidase and CCL18 cannot be used as biomarkers of Gaucher cells in mice, gpNMB offers a very attractive alternative in this respect. Fundamental investigations on pathophysiology and therapy in GD mouse models may profit from determination of gpNMB as biomarker for Gaucher cells.

In conclusion, our finding of markedly elevated plasma gpNMB levels in symptomatic Gaucher patients warrants further investigations regarding its applicability in the clinical management of Gaucher disease as well as its role in the peculiar pathophysiology of the disorder.

## Author contributions

GK, WW and MvE designed, performed research, and analyzed the data. WD‐K, RO, PG, MV, JN, TG, WK, RGB and JDL performed research and analyzed data. JPCV, TC, EP and MTM developed, produced, and provided key experimental reagents. JMA and MvE wrote manuscript.

## Supporting information


**Fig. S1.** LC‐MS^E^ of Gaucher spleen lysate.
**Fig. S2.** Correlation of plasma gpNMB and β‐hexosaminidase.
**Fig. S3.** Analysis of gpNMB in LIMP2^−/−^ mice liver and plasma.

**Table S1.** Individual values of gpNMB (ng·mL^−1^) in GD (t0) serum and after 1 year (t1) of substrate reduction therapy.
