# Supplementary figures and images for: Elevation of glycoprotein nonmetastatic melanoma protein B in type 1 Gaucher disease patients and mouse models
Source: FEBS Open Bio. 2016 Jul 30;6(9):902–13. doi: 10.1002/2211-5463.12078 (PMC5011488; doi:10.1002/2211-5463.12078)

Suppl Fig 1

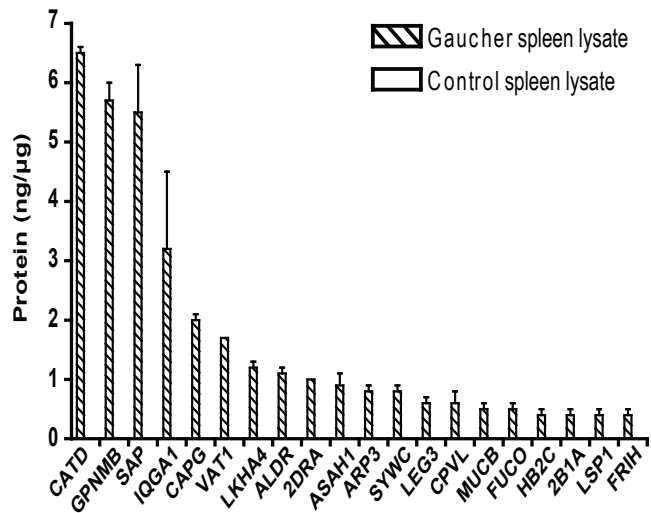

Suppl Fig 2

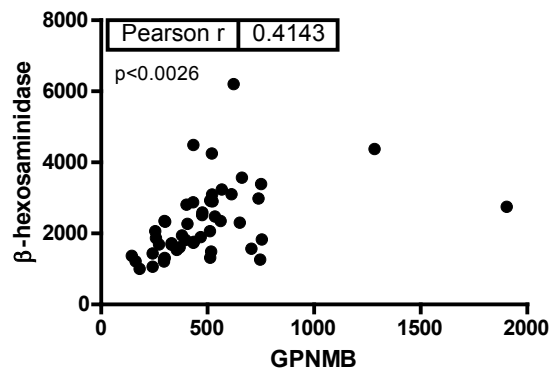

Suppl Fig 3

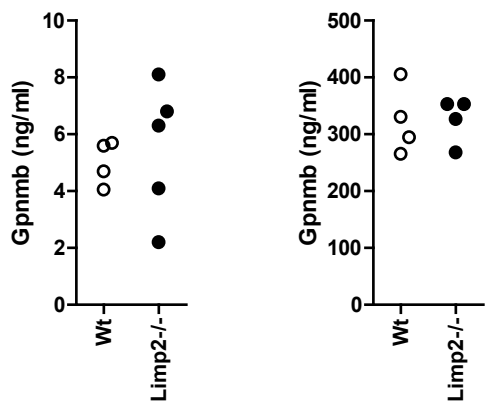

Supplement: Supplementary file 1 — Fig. S1. LC‐MSE of Gaucher spleen lysate. Fig. S2. Correlation of plasma gpNMB and β‐hexosaminidase. Fig. S3. Analysis of gpNMB in LIMP2−/− mice liver and plasma. [file FEB4-6-902-s001.pdf]
