# Supplementary material for: Elevation of glycoprotein nonmetastatic melanoma protein B in type 1 Gaucher disease patients and mouse models
Source: FEBS Open Bio. 2016 Jul 30;6(9):902–13. doi: 10.1002/2211-5463.12078 (PMC5011488; doi:10.1002/2211-5463.12078)
Supplement: Supplementary file 2 — Table S1. Individual values of gpNMB (ng·mL−1) in GD (t0) serum and after 1 year (t1) of substrate reduction therapy. [file FEB4-6-902-s002.docx]

**Supplemental table I**

| **gpNMB (t0)** | **gpNMB (t1)** | **% gpNMB correction** |
| --- | --- | --- |
| 297 | 130 | 56 |
| 433 | 201 | 54 |
| 392 | 233 | 41 |
| 330 | 136 | 59 |
| 516 | 83 | 84 |
| 511 | 285 | 44 |
| 180 | 70 | 61 |
| 162 | 59 | 64 |
| 396 | 70 | 82 |
| 751 | 398 | 47 |
| 295 | 167 | 43 |
| 379 | 191 | 50 |
| 432 | 92 | 79 |
| 435 | 249 | 43 |
| 534 | 319 | 40 |
| 1283 | 464 | 64 |
| 660 | 323 | 51 |
| 355 | 166 | 53 |
| 512 | 97 | 81 |
| 242 | 183 | 24 |
| 515 | 332 | 36 |
| 521 | 176 | 66 |
| 474 | 307 | 35 |
| 433 | 276 | 36 |
| 651 | 50 | 92 |
| 561 | 222 | 60 |
| 400 | 283 | 29 |
| 257 | 70 | 73 |
| 467 | 131 | 72 |
| 404 | 189 | 53 |
| 330 | 115 | 65 |
| 566 | 257 | 55 |
| 794 | 142 | 82 |
| 300 | 141 | 53 |
| 848 | 653 | 23 |
| 1903 | 299 | 84 |
| 519 | 230 | 56 |
| 612 | 211 | 66 |
| 298 | 239 | 20 |
| 512 | 257 | 50 |
| 295 | 144 | 51 |
| 242 | 75 | 69 |
| 368 | 241 | 35 |
| 405 | 131 | 68 |
| 272 | 65 | 76 |
| 305 | 287 | 6 |
| 705 | 188 | 73 |
| 738 | 147 | 80 |
| 622 | 145 | 77 |
| 523 | 139 | 73 |
| 254 | 140 | 45 |
| 929 | 205 | 78 |
| 754 | 553 | 27 |
| 475 | 266 | 44 |

Individual values of gpNMB (ng/ml) in GD (t0) serum

and after 1 year (t1) of substrate reduction therapy.
